# The Role of Astrocytes and Alpha-Synuclein in Parkinson’s Disease: A Review

**DOI:** 10.3390/neurosci5010005

**Published:** 2024-03-08

**Authors:** David Brash-Arias, Luis I. García, César Antonio Pérez-Estudillo, Fausto Rojas-Durán, Gonzalo Emiliano Aranda-Abreu, Deissy Herrera-Covarrubias, Donaji Chi-Castañeda

**Affiliations:** 1 Doctorado en Investigaciones Cerebrales, Instituto de Investigaciones Cerebrales, Universidad Veracruzana, Xalapa 91190, Mexico; david.brash.arias@gmail.com; 2 Instituto de Investigaciones Cerebrales, Universidad Veracruzana, Xalapa 91190, Mexico

**Keywords:** astrocytes, Parkinson’s disease, α-syn, neurodegeneration, therapies, neurodegenerative disease

## Abstract

The search for new therapies to reduce symptoms and find a cure for Parkinson’s disease has focused attention on two key points: the accumulation of alpha-synuclein aggregates and astrocytes. The former is a hallmark of the disease, while the latter corresponds to a type of glial cell with an important role in both the prevention and development of this neurodegenerative disorder. Traditionally, research has focused on therapies targeting dopaminergic neurons. Currently, as more is known about the genetic and molecular factors and the neuroglial interaction in the disease, great emphasis has been placed on the neuroprotective role of astrocytes in the early stages of the disease and on the astrocytic capture of alpha-synuclein under both physiological and pathological conditions. This review aims to analyze the contribution of alpha-synuclein and astrocytes to the development and progression of Parkinson’s disease, as well as to evaluate recent therapeutic proposals specifically focused on synucleopathies and astroglial cells as potential therapies for the disease.

## 1. Introduction

Parkinson’s disease (PD) was first described by James Parkinson in “An Essay on the Shaking Palsy”, in which he hoped for a possible cure, or at the very least, a way to halt the disease’s progress [1]. Over 200 years later, PD remains an incurable neurodegenerative disorder characterized by progressive motor deficits, such as tremors, muscular stiffness, postural instability, and bradykinesia [2].

Globally, it is the second most common neurodegenerative disease, with the highest disability, prevalence, and mortality rates among all neurodegenerative diseases [3]. In Mexico, a prevalence of 6.7 cases per 100,000 habitants was reported in 2014, with an estimated increase to 14.9 cases per 100,000 habitants by 2023 [4].

The clinical manifestations of PD result from the selective degeneration of dopaminergic neurons in the substantia nigra pars compacta (SNpc), leading to abnormally low dopamine (DA) levels in the striatum, along with non-motor symptoms such as sleep disturbances, dementia, sensory abnormalities, and autonomic dysfunction [5,6]. This depletion of striatal DA is accompanied by the appearance of Lewy bodies (LBs), which are intracytoplasmic inclusions and a distinctive hallmark within surviving neurons [7].

The etiology of PD is largely unknown, but it is understood to be a multifactorial disease influenced by environmental factors, aging, and genetics [8]. Specific genetic factors that play a significant role in the risk of developing this disease can be identified in subsets of PD patients [9]. In 1996, Polymeropoulos et al. identified a mutation in the *alpha-synuclein* gene (*SNCA*), associated with rare cases of autosomal dominant PD. Although families with such mutations are uncommon, this discovery revealed that alpha-synuclein (α-syn) is an important component of LBs, even in sporadic PD patients [10,11].

It has been demonstrated that astrocytes play an essential role in neurodegenerative diseases, both in early stages and during the disease’s progression [12,13,14]. Astrocytes are non-neuronal cells crucial for the homeostatic balance of the central nervous system (CNS), maintaining ionic and metabolic homeostasis, neurotransmission, and synaptic pruning, as well as providing trophic and metabolic support to neurons, among other functions [15,16]. *Post mortem* studies of PD-diagnosed patients have reported that astrocytes accumulate α-syn during the disease’s course [13,14]. Altered α-syn released by axonal terminals in surrounding synapses is absorbed by astrocytes, supporting the hypothesis of α-syn propagation through neuron–astrocyte interactions [13,17].

The fact that astrocytes accumulate α-syn aggregates has sparked interest in recent years to develop research lines focused on therapies targeting astroglia to generate disease-modifying or -reducing effects through the design of immunologically based biotherapies and the replacement of functional astrocytes, among other approaches [18,19].

In this review, we summarize the pathophysiological mechanisms of astrocytes in PD, their interaction with α-syn protein, and current perspectives on the use of various novel therapies focused on both astroglia and α-syn in PD.Principio del formulario.

## 2. Astrocytic Processes in Parkinson’s Disease

In most pathological conditions of the brain, astrocytes often undergo changes in both their morphology and function, a process referred to as reactive astrogliosis. This process has the following key characteristics: hypertrophy and the increased expression of intermediate filaments, primarily glial fibrillary acidic protein (GFAP) [20].

Particularly, reactive astrocytes respond to acute cellular stress and work to limit damage to the CNS, but chronic reactive astrogliosis can result in the sustained production of reactive oxygen and nitrogen species (ROS/RNS), as well as the release of proinflammatory molecules, which promotes neuronal injury and neurotoxicity [21]. 

Although the exact signaling pathways by which reactive astrogliosis promotes dopaminergic neuronal death are not fully understood, it is believed that astrogliosis can have both beneficial and detrimental effects on dopaminergic neurons [22]. An increasing amount of evidence has revealed that reactive astrocytes produce proinflammatory cytokines (TNF-α, IL-1β, IL-6, and interferon-γ), which mark the initiation of neuronal apoptosis through the activation of caspases 3 and 8, as well as cytochrome c [23,24]. Furthermore, reactive astrocytes release nitric oxide into the extracellular space, causing increased lipid peroxidation, mitochondrial damage, and DNA strand breaks and eventually leading to neuronal injury and death [25].

In 2017, it was discovered that neuroinflammation and ischemia induced two types of reactive astrocytes, which were named A1 (harmful) and A2 (protective) due to their nomenclature (analogous to the M1 and M2 macrophage and microglia paradigm) [26]. In this report, the authors demonstrated in an in vivo mouse model with acute CNS injury that A1 astrocytes are induced by activated microglia and lose their normal functions as astrocytes although they acquire a new neurotoxic function, leading to neuronal death and a decreased phagocytic capacity. Likewise, through a co-culture of control astrocytes and A1 astrocytes from retinal ganglion cells, it was found that astrocytes generate a soluble toxin that rapidly kills subsets of CNS neurons and mature oligodendrocytes and induce the ability to promote neuronal survival and growth [26].

While evidence shows that A1 astrocytes lose their neuroprotective functions and contribute to neuronal death, A2 astrocytes produce antioxidant molecules in response to oxidative stress and protect dopaminergic neurons in rat PD models, both *in vitro* and *in vivo* [27]. 

Several studies on the detrimental effects of A1 astrocytes on neurons have been reported in recent years; however, the protective effects of A2 astrocytes remain to be fully explored. So far, genetic profiling has shown that A2 astrocytes induced by a middle cerebral artery occlusion in a mouse model upregulate beneficial neurotrophic factors and inflammatory factors, such as cardiotrophin-like cytokine factor 1 (CLCF1), hypoxia-inducing factor (HIF), IL-6, IL-10, and thrombospondins, which promote neuronal survival and remodeling [16,28].

Additionally, it has been demonstrated in primary mouse astrocytes that the overexpression of the chemokine-like signaling protein prokineticin-2 (PK2) induces the A2 astrocytic phenotype, as well as the upregulation of key protective genes and A2 reactivity markers (PTX3, SPHK1, TM4SF1, and Nrf2). This response was accompanied by an increase in glutamate/aspartate transporter (GLAST) expression and glutamate uptake, indicating that A2 astrocytes can reduce extracellular glutamate through the positive regulation of GLAST, preventing neuronal excitotoxicity [29].

Several reports indicate that reactive astrocytes contribute to the development and progression of neurodegenerative diseases through pathological processes involving neuroinflammation, neuroprotection deficits [30,31], aberrant neurotransmitter uptake [32], and inefficient gliotransmission [33].

Due to the fact that glial-derived neurotrophic factor (GDNF) promotes the survival and differentiation of dopaminergic neurons [34] and that the blood–brain barrier (BBB) is compromised in PD patients [35,36], it is reasonable to assume that the impairment and loss of astrocyte functions are implicated in the onset and progression of PD.

It is well documented that astrocytes play a fundamental role in maintaining the BBB. These cells modify the formation of tight junctions in endothelial cells through the release of regulatory factors such as transforming growth factor-alpha (TGF-α) and GDNF, controlling microvascular permeability through proper astrocyte–endothelial interactions [37]. The disruption of the BBB is thought to contribute to the progression of PD since increased BBB permeability is related to the infiltration of inflammatory mediators into the CNS, promoting microglial activation and dopaminergic neurodegeneration [38]. In addition to these findings, Gray and colleagues reported increased BBB permeability in the putamen of PD patients [36].

Furthermore, the dysfunction of the astrocytic glutamate transporter EAAT2/GLT-1 leads to extracellular glutamate accumulation, generating excitotoxicity due to the overstimulation of excitatory amino acid receptors. Recently, a study demonstrated that GLT-1 deficiency in the substantia nigra pars compacta in mouse SNpc induced Parkinsonian phenotypes, progressive motor deficits, and DA neuronal death. This suggest that GLT-1 transporter dysfunction also contributes to the development and progression of PD [39].

## 3. Alpha-Synuclein

In the mid-1990s, Polymeropoulos and colleagues identified the gene responsible for PD through a linkage analysis and association analysis for familial and sporadic PD, respectively [10,40]. The *SNCA* gene, which encodes the alpha-synuclein (α-syn) protein, was the first gene identified in familial PD, and subsequently, the presence of aggregates of this protein was described for different forms of the disease, including sporadic and autosomal dominant PD [10,41].

The α-syn protein is a 140-amino-acid protein that comprises an N-terminal region that assumes an α-helical secondary structure upon membrane binding, a non-amyloid-component hydrophobic domain that can adopt a β-sheet conformation, promoting protein aggregation in its monomeric form, and a negatively charged C-terminal domain [42]. This protein is abundant in the CNS and is primarily localized in presynaptic terminals [43].

The physiological function of α-syn remains unclear. Some evidence suggests that physiological levels of this protein play a role in regulating the presynaptic function of the SNARE complex, which is responsible for neurotransmitter release [44]. Additionally, α-syn is known to have functions associated with regulating vesicular and membrane dynamics [45,46]. Conversely, the excessive accumulation of α-syn has been linked to PD. 

The triplication of the *SNCA* gene locus leads to increased α-syn levels, which has been associated with a higher risk of developing PD [11]. The mutations in α-syn (A53T, A30P, and E46K) also increase the probability of developing PD since they alter the secondary structure of this protein, promoting its neuronal and astrocytic aggregation [47]. Furthermore, alleles within a *Rep1* polymorphic region, 10 kB upstream of the *α-synuclein* gene promoter [48], have been associated with increased *α-syn* mRNA expression in human and mouse neurons [49,50] as well as in the substantia nigra in humans [51]. These findings suggest that increased *α-syn* mRNA concentrations and α-syn protein levels are triggering factors for the development and progression of PD [52].

Under physiological conditions, α-syn can bind to the lipid membrane to promote the assembly of the SNARE complex and the formation of stable tetramers resistant to physiological aggregation processes [53]. When the balance between α-syn production and clearance is disrupted, this protein aggregates and unfolds into oligomers, then amyloid fibrils, and finally into LBs [52]. The α-syn aggregates exhibit diverse structures, ranging from soluble oligomeric ring-shaped, rope-shaped, or spherical forms (protofibrils) to insoluble fibrils [54,55,56]. These fibrils are thought to form the basis of Lewy bodies, although it is controversial whether the smaller protofibrils or the larger amyloid fibrils are the toxic α-syn species that cause neuronal cell death [57,58]. Among the multiple ways by which α-syn oligomers can induce cytotoxicity are mitochondrial damage, endoplasmic reticulum stress, synaptic impairment, excitotoxicity, neuroinflammation, proteostasis loss, and cell apoptosis [59].

The ubiquitin–proteasome system (UPS) and the lysosomal autophagy pathway (ALP) are the main pathways for eliminating overexpressed or misfolded proteins in cells to maintain protein homeostasis. It has been reported that the UPS is the primary pathway for degrading α-syn, although once saturated, the ALP also participates in the degradation process [60].

The reciprocal interaction between α-syn and the proteasome function suggests a self-perpetuating process in which permanently elevated levels of α-syn impair the UPS, which in turn may lead to the increased accumulation of α-syn. The detailed molecular mechanism by which α-syn is degraded by the proteasome is still not known. There is evidence in cell cultures that degradation can occur through a proteasome-dependent, ubiquitin-independent pathway [61,62,63].

In 2011, in a human α-syn WT transgenic mouse model, it was found that the UPS degrades α-syn under conditions with an increased endogenous α-syn load and that in contrast, autophagy is used to degrade α-syn only when intracellular levels of α-syn are elevated, providing a link between the proteasome, autophagy, and synucleopathies, being one of the first pieces of evidence in an *in vivo* model of protein loading and the pathways recruited to maintain homeostasis [60].

Recently, it has been reported that the down-regulation of the UPS and ALP leads to the accumulation of α-syn oligomers, which in turn inhibit the protein elimination process. By promoting the removal of α-syn oligomers, several research groups agree that targeting the signaling pathways involved in both systems can be an efficient way to restore proteostasis, becoming a potential and promising therapeutic target for PD [59,60,64,65,66].

Collectively, the evidence suggests that all misfolded proteins in neurodegenerative diseases show prion-like seed effects, including α-syn. Findings have shown the detection of α-syn-positive LBs in grafts from PD patients who received a transplant of embryonic midbrain cells, indicating the existence of the host-to-graft transfer of α-syn pathology and prion-like behavior [59].

### Alpha-Syn: Its Prion-like Spreading and Presence in the Peripheral and Enteric Nervous System

The discovery that misfolded α-syn exhibits properties similar to those of prions has attracted interest in our understanding the progression of PD according to the Braak staging system [9], which classifies the progression of PD into six stages. The first stages (one and two) are pre-symptomatic, characterized by the loss of non-motor functions, such as the loss of one’s sense of smell. At these stages, there may be more prevalent Lewy neurites than LBs, and the brain stem is the most affected. In the intermediate stages (three and four), patients lose motor functions and develop bradykinesia and rigidity. At this point, the disease passes to the striatum and LBs are formed. In the final stages (five and six), patients have all the other symptoms, the disease progresses to other parts of the brain, and there may be neuronal losses [67].

These findings suggest the propagation of α-syn from one brain region to another, a conclusion that is supported by studies in which fetal mesencephalic neurons transplanted into PD patients also developed a Lewy pathology [68,69]. Additionally, the administration of exogenous preformed fibrils of α-syn (PFF) to cultured neurons and intracerebral injections in wild-type (WT) mice led to the aggregation of endogenous α-syn and the subsequent propagation of this pathology [70,71,72].

Particularly, using aggregates isolated from brains of LB and PD patients, Recasens and coworkers seeded LB-enriched fractions containing α-syn. The result was the initiation of progressive neurodegeneration following a pattern similar to the Braak staging system, in both mice and primates [73]. On the other hand, by injecting PFF into WT mice or *SNCA*^−/−^ mice, it was evaluated whether pathological α-syn can be transported through the vagus nerve and if endogenous α-syn is required for the propagation of pathological α-syn. It was found that α-syn can propagate from the gastrointestinal tract through the vagus nerve to the brain, supporting the Braak staging hypothesis [74].

These findings have led to the conclusion that α-syn pathology in PD is not limited to the CNS but also involves the peripheral and enteric nervous systems [75]. Over the past two decades, largely based on Braak and colleagues’ findings, it has been proposed that PD may be caused by a pathogen that enters the body through the nasal cavity, is subsequently swallowed, and reaches the intestine, initiating Lewy pathology in the olfactory bulb and the digestive tract, explaining the simultaneous occurrence of the disease in both regions, as per the Braak staging system [67,76].

Despite the fact that Braak’s staging hypothesis in PD has been clinically validated both *in vivo* and *in vitro*, there is still a lack of understanding regarding its underlying molecular mechanisms. Furthermore, it is necessary to consider some documented inconsistencies [77]. For example, in the study published by Braak and colleagues in 2002, only 30 of 413 cases of Lewy neurites in the dorsal motor nucleus of the vagus nerve were used to provide the basis of the stratification scheme. Likewise, no Lewy bodies were found in the SNpc of 30 of the sporadic PD patients [77]. Another study suggests that the Braak system does not allow for the classification of nearly 50% of PD cases. Zaccai and collaborators reported that only 51% of the 208 cases of autopsies in patients with a diagnosis of PD followed the Braak pattern [78]. Other reports have provided evidence invalidating a smooth and predictable rostro-caudal progression of the synucleinopathy abnormality in the brains of people with PD [79]. 

Although the aforementioned are studies that cast doubt on the Braak staging system, there is evidence with important case statistics that allow us to elucidate patterns of disease development, although more clinical, molecular, and pathological studies are required to strengthen this hypothesis

## 4. Astrocytes and **α**-Syn

Astrocytes greatly contribute to neuronal survival through numerous mechanisms, such as the secretion of neurotrophins and antioxidants, the clearance of α-synuclein, glutamate metabolism, fatty acid metabolism, and the transfer of healthy mitochondria to neurons [80]. However, the effects of astrocytic protection can be heterogeneous and depend on the brain region in which they are located and the loss of homeostatic balance of the CNS [81]. In contrast, reactive astrocytes are those that have undergone various cellular, molecular, and functional changes in response to injury or neurodegenerative diseases [80]. 

One of the hallmarks of PD is the formation of α-syn deposits. Several studies have demonstrated that astrocytes promote the formation and propagation of these protein deposits [13,14,82,83,84]. The first piece of evidence of the close relationship between α-syn pathology and astrocytes was through the analysis of post mortem brain tissue from patients with PD. Particularly, Wakabayashi et al. revealed that α-syn immunoreactive inclusions are frequently found in SNpc astrocytes of PD patients [14].

α-synuclein aggregates are primarily found in astrocytes [13,14], and the accumulation of α-syn in these cells occurs through intercellular transfer [85] as the astrocytes capture α-syn released by axonal terminals [17]. *In vitro* and *ex vivo* studies have shown that α-syn can be transmitted from neuron to neuron, astrocyte to astrocyte, and bidirectionally between neurons and astrocytes, although transmission from neurons to astrocytes or between astrocytes is much more efficient [86]. Extensive uptake leads to the incomplete and inefficient degradation of α-syn oligomers due to overloading the lysosomal degradation pathway, resulting in the formation of intracellular astrocytic protein deposits and mitochondrial damage [87].

Toxic α-syn has been shown to induce mitochondrial damage and increased mitochondrial fragmentation, as well as affect mitophagy in neuronal cells [87,88,89,90]. Also, in human primary astrocytes, α-syn has been suggested to locate to the mitochondria and cause reduced oxygen consumption [91]. In a study of primary astrocytes from mice infected with α-syn oligomers, mitochondrial damage with fragmentation patterns was confirmed, coupled with reduced mitochondrial functionality with abnormally high levels of reactive oxygen species (ROS), generating oxidative stress and neuronal death [87].

It has been reported that neuronal loss and the presence of cytoplasmic inclusions in neuronal cells are accompanied by astrogliosis [27]. In 2007, Braak and his team reported that many α-syn-positive astrocytes appeared in stages 4 to 6 of the Braak PD stages, concentrating in the prosencephalon, specifically in the amygdala, thalamus, septum, striatum, claustrum, and cerebral cortex [13].

The overexpression of the mutant *SNCA* gene in primary astrocytes from a mouse model disrupted the normal functioning of these cells, impaired BBB permeability, and disrupted the homeostasis of glutamate uptake by astrocytic transporters (GLAST and GLT-1) [92]. On the other hand, a study using human brain homogenates from PD and LB patients demonstrated that α-syn is captured and propagated from astrocytes to neurons, causing neuronal death [93].

Studies on astrocytes derived from induced pluripotent stem cells (iPSCs) of familial PD patients have demonstrated that α-syn accumulation in astrocytes directly correlates with its toxicity to neurons [92,94]. Pathological α-syn contributes to the formation of A1 astrocytes, which prevent microglia-induced activation mediated by α-syn. In a sporadic PD mouse model, it was demonstrated that using agonists of GLP-1R (glucagon-like peptide) expressed on astrocytic membranes to prevent the conversion of astrocytes to a neurotoxic A1 phenotype mediated by microglia reduced the disease’s progression [95].

The transmission of α-syn from neuron to astrocyte, followed by its accumulation and the formation of intra-astrocytic deposits, generates neuroinflammation and contributes to PD neurodegeneration. Astrocytes with accumulated α-syn produce pro-inflammatory cytokines, such as IL-1, IL-6, and TNF-α, as well as chemokines CXCL1 and CX3CL1 [17]. Additionally, a 2010 study showed that the expression of the α-syn A53T mutation related to PD, selectively in astrocytes, disrupted astrocytic functions related to glutamate uptake and BBB regulation, leading to microglial activation, inflammatory responses, and dopaminergic neurodegeneration in mice [92].

Recently, a report identified a link between the molecular circadian axis (BMAL1-BAG3 axis) in astrocytes and α-syn aggregation. It has been shown that the silencing of the *Bmal1*^−/−^ clock gene in astrocytes was enough to prevent α-syn pathology *in vivo* and induce the activation of these cells. This response was associated with the increased astrocytic phagocytosis of α-syn by BAG3 (a macroautophagy chaperone) [96].

The mechanisms underlying α-syn internalization by astrocytes are thought to be different from neuronal mechanisms. In neurons, such internalization can occur in different ways, such as the interaction with heparin sulfates on the cell surface [97], Lag3 receptors [98], or the sodium–potassium transport subunit ATPase β3 [99]. However, in astrocytes, it remains unclear, largely because they possess a unique interactome. Since astrocytic receptors that specifically bind to α-syn oligomers have not been identified so far, the development of therapeutic targets based on blocking astrocytic receptors continues to be studied [100].

Although the findings mentioned above demonstrate that both dysfunctional astrocytes and reactive astrogliosis contribute to PD pathogenesis and progression, it has been reported that in the early stages of the disease, astrocytes play a protective role in clearing α-syn deposits. However, this mechanism becomes compromised and inefficient as α-syn deposition persists [101]. Therefore, a dual role of astrocytic dysfunction in PD pathophysiology is currently proposed with a potential therapeutic role for these cells in PD and synucleopathies.

## 5. New Therapies Targeting Astrocytes and **α**-Syn for PD Treatment

### 5.1. Pharmacotherapy Targeting Astrocytes in PD

In the present day, multiple studies have demonstrated neuroprotective approaches focused on astrocytes in Parkinson’s disease models [27,102,103]. Particularly, the activation of the Nrf2 signaling pathway in astrocytes provides neuroprotection by producing antioxidant molecules [104]. In a complex with small Maf proteins, Nrf2 binds to antioxidant-responsive elements (AREs) that induce the transcriptional activation of downstream genes encoding phase II antioxidant and detoxifying enzymes [105,106]. It has been reported that in PD, there is a dysfunction of this pathway, and its overexpression can increase the ability of astrocytes to reduce oxidative stress and inflammatory responses [107].

In addition to these findings, the overexpression of Nrf2 in astrocytes delayed the onset of motor dysfunction and α-syn aggregation in mutant α-syn transgenic mice (A53T) [77]. Several studies have shown that curcumin, sulforaphane, and resveratrol can activate Nrf2 and exert neuroprotective effects against oxidative stress in animal models of neurodegenerative disorders [108,109,110].

Previous studies demonstrate that the astrocyte-specific overexpression of Nrf2 is able to reduce chemically mediated neurotoxicity in models of PD and Huntington’s disease [111,112]. Another report showed that Nrf2 deficiency can exacerbate α-syn aggregation in mice using an adeno-associated viral vector expressing human α-syn [113]. In a Drosophila model with an overexpression of α-syn and genetic increase in Nrf2, locomotor activity was restored [114]. The findings related to Nrf2 make it clear that this protein represents a key point for the development of new therapies targeting astrocytes in patients with PD.

On the other hand, preclinical strategies specifically targeting astrocytes have been proposed. For instance, the glucagon-like peptide 1 receptor (GLP1R) agonist, NLY01, has demonstrated efficacy in multiple models of neurodegenerative proteinopathies by modulating mitochondrial biogenesis, microglial inflammation, and improved autophagy processes, as well as the clearance of aggregated proteins. NLY01 regulates NF-ΚB-mediated inflammatory signaling, located downstream of the GLP-1R/PI3K/AKT pathway [115].

Recently, it was determined that NLY01 blocks the pathological microglial activation induced by α-syn, also inhibiting the induction of the inflammatory mediator, IL-1α, TNFα, and C1q, preventing astrocyte reactivity. In two PD mouse models associated with α-synuclein pathology, NLY01 injected subcutaneously showed strong anti-PD efficacy by blocking microglial activation and reactive astrocyte conversion, obtaining synergistic anti-inflammatory and neuroprotective effects, as well as an improvement in cognition and neurobehavior without any adverse effects [116].

In the study conducted by Isooka and colleagues, it was demonstrated that rotigotine, a dopaminergic anti-Parkinsonian drug and partial agonist of 5-HT1A receptors, induced astrocyte proliferation through the Nrf2 signaling pathway, reducing dopaminergic neurodegeneration in Parkinsonian mice. These results support the idea that it is necessary to continue developing pharmacological agents targeted at 5-HT1A receptors in astrocytes as a promising therapeutic strategy for neuroprotection in PD treatment [103].

The astrocytic glutamate transporter GLT-1 is a promising therapeutic target as various drugs positively regulate it, acting as potential neuroprotective agents by preventing excitotoxicity. An example is ceftriaxone, a β-lactam antibiotic that increases GLT-1 expression, exerting neuroprotective effects on dopaminergic neurons and improving motor deficits in rat models of PD [117]. Furthermore, ceftriaxone has been reported to improve cognition and prevent hippocampal neurodegeneration in an animal model of PD-related dementia [118].

The use of antidepressant drugs such as fluoxetine has led to improvements in behavioral and neuropathological deficits in transgenic mouse models with α-synucleinopathy [119]. Additionally, sertraline has been shown to decrease α-syn uptake by neurons and oligodendrocytes, significantly reducing motor dysfunction, oxidative stress, mitochondrial damage, and neuroinflammation in rat models of Parkinson’s disease [120,121].

Given the aforementioned findings, the role of antidepressants and other drugs identified as possible pharmacological therapies for PD is promising due to their potential neuroprotective role in various forms of the disease.

### 5.2. Conversion of Astrocytes to Dopaminergic Neurons and Therapies with Functional Astrocytes

New therapies that use functional astrocytes to replace dysfunctional ones and convert them to dopaminergic neurons offer a promising possibility since it has been suggested that they can slow down and even halt the progression of PD [19].

In 2017, human astrocytes were successfully reprogrammed into induced dopaminergic neurons with mesencephalic neuronal markers and electrophysiological properties *in vitro*. In PD-modeled mice using the neurotoxin 6-OHDA, reprogrammed dopaminergic neurons from adult striatal astrocytes were shown to be functional and improved signs of motor dysfunction, such as bradykinesia [122].

In a stable neurotrophic brain environment, ventral mesencephalic (VM) co-cultured astrocytes were induced to differentiate into dopamine neurons, which displayed higher levels of neuronal maturity, increased mesencephalic-specific marker levels, and greater resistance to cytokines compared to control cultures [123].

Furthermore, it has been demonstrated that the cellular reprogramming of astrocytes into dopaminergic neurons in the striatum of adult mice, through the removal of the RNA-binding protein PTB (PTBP1) using CRISPR-Cas9, reduced motor deficits in 6-OHDA-induced PD mice. In that study, it was found that the suppression or elimination of PTBP1 resulted in the conversion of mesencephalic astrocytes into dopaminergic neurons, restoring dopamine levels [124].

Cell transplantation therapies in in vivo models have generated promising evidence in PD treatment. In a hemi-Parkinsonian rat model, it was demonstrated that astrocyte co-transplantation enhanced the therapeutic effects of neural progenitor cell transplantation up to 6 months post-transplantation [88]. Astrocyte grafting into the SNpc in elderly Parkinsonian mice after the onset of motor symptoms showed the reversal of nigrostriatal neuronal loss, increased dopamine uptake, and improvements in motor deficits [125].

Recently, it was shown that cultured astrocytes from the VM, a brain region primarily affected in PD, substantially improve neuronal pathology with a high presence of α-syn in hemi-Parkinsonian mice. The VM astrocytes efficiently regulated a series of proteostasis processes associated with the formation, transmission, and clearance of toxic α-syn aggregates. Following transplantation, the VM astrocytes effectively eliminated pathological α-syn accumulation and dopaminergic neuron degeneration [126]. These findings propose cultured and transplanted VM astrocytes as a new therapy for PD to treat endogenous synucleinopathy in the mesencephalon, preventing the transmission of toxic α-syn from the host to the graft through a stem-cell-based therapeutic approach [19,126].

### 5.3. Immunological Biotherapies Targeting α-Syn

There are currently two therapeutic approaches being tested in PD patients: small-molecule compounds that inhibit the accumulation of α-syn and anti-α-syn immunotherapy, both active and passive [127]. However, neither of these approaches are directly targeted at astrocytes, as it has not yet been determined how to harness the immune system to achieve beneficial results based on these cells [18].

#### 5.3.1. Small-Molecule Compounds

Some small-molecule compounds have been shown to suppress the neurotoxicity of α-syn oligomers through various mechanisms. Among them is squalamine, a natural compound with anticancer and antiviral properties capable of blocking the α-syn aggregation process by inhibiting the interaction between oligomers and lipid membranes [128]. In an *in vitro* model, the effects of baicalein, a natural flavonoid compound believed to prevent the formation of α-syn oligomers, were studied, and it was found to reduce motor deficits in a mouse model of PD [129]. Epigallocatechin gallate, a natural component of green tea, showed neuroprotective effects by promoting the production of non-toxic oligomers, facilitating the pathological oligomers’ reduction in toxic fibril formation [130].

#### 5.3.2. Antibodies Targeting α-Syn

One of the most challenging strategies in recent years for PD treatment is the use of antibodies targeting α-syn. Various research groups have reported evidence of neuroprotection through passive (antibody-based) or active (vaccination-based) immunotherapy. However, the specific α-syn species and conformation that should be targeted in antibody design should be considered, as some α-syn species may be protective, and their removal could accelerate the disease process [131].

There are several antibodies targeting α-syn oligomers, which do not recognize linear epitopes but rather conformational ones, so they only recognize particular conformations [132]. The mAb47, a selective antibody for α-syn oligomers, enters human neuroglioma cells through Fcγ receptors and neutralizes toxic protein aggregates [133]. Transgenic PD mice treated with this antibody, which preferentially binds to oligomeric conformations, show a reduced intracellular aggregation of α-syn and lower extracellular oligomer levels [134]. Three types of antibodies, Syn-O1, -O2, and -F1, were able to recognize different α-syn species and decrease α-syn oligomer accumulation, slowing down neurodegeneration in an *in vivo* PD mouse model [135].

Although the design of these antibodies is still in the early stages of development, findings in *in vivo* models suggest that they are capable of neutralizing and depolymerizing α-syn oligomers, making their development a viable therapeutic strategy.

## 6. Conclusions and Future Prospects

Astrocytes are the most abundant glial cells in the brain. Despite the fundamental role of their multiple functions in maintaining CNS homeostasis, their involvement in the etiology and progression of neurodegenerative diseases, including PD, is not well documented. The formation of α-syn aggregates and deposits is a hallmark of PD, and both the accumulation and elimination of this protein largely depend on astrocytes.

The capture and elimination of α-syn aggregates appear to be a physiological mechanism of astrocytes. However, in the pathophysiology of PD, the overload of this protein leads to misfolding, resulting in an oligomeric conformation that saturates these mechanisms, making them inefficient. This, in turn, triggers a series of inflammatory, toxic, and apoptotic processes that promote disease progression. At this point, the bivalent role of astrocytes must be emphasized, as they have both harmful and beneficial roles, at least at the onset of PD.

PD is a complex and heterogeneous disease influenced by environmental, genetic, and age-related factors. It is necessary to evaluate the individual and/or family profiles of patients to allow for the classification and stratification of each case and personalize their available treatment options.

Previously, therapies were focused on the selective loss of DA neurons and the replenishment of dopamine levels. Nowadays, evidence suggests a new approach in studies focused on maintaining the functionality of astrocytes to prevent neurodegeneration and improve neuronal survival, considering the heterogeneity of this group of cells in terms of both function and distribution in the CNS, along with findings directly linking them to synucleinopathies (Table 1).

New stem cell therapy approaches, using cultured VM astrocyte transplantation in situ, have recently emerged as a new therapeutic option for treating α-syn in PD, not only due to the benefits of physiological astrocyte processes but also due to the low or negligible risk of transmitting toxic α-syn from the host to the transplanted cells.

Since it is very likely that astrocytes play a protective role in nigrostriatal DA neurons during early neurodegeneration, therapeutic approaches using functional astrocytes to replace dysfunctional ones and in part, convert them into dopaminergic neurons, are promising for slowing down and even halting the progression of PD. Pharmacological therapies targeting glutamate transporters and the modification of astrocytic signaling pathways, as well as the use of specific antibiotics and antidepressants, have reduced signs of deterioration and increased neuroprotection in *in vivo* mouse PD models. Biochemical, genomic, and imaging biomarkers in patients are still required to conduct accurate clinical trials and develop immunological biotherapies, both active and passive.

The results of recent experimental research conclude that it is essential to continue focusing on the function and dysfunction of astrocytes to correct the damage caused by α-syn in PD. Without a doubt, astrocytes are promising therapeutic targets, although further studies are needed to understand how these cells contribute to different forms of PD for a better understanding of the disease and to enrich current and future therapeutic approaches.

## Figures and Tables

**Table 1:** Novel therapies targeting astrocytes or α-syn in Parkinson’s disease.

**Therapies Targeting Astrocytes**				
**Therapies**	**Effects in Astrocytes**	**Findings in the PD**	**Experimental Approach**	**References**
*Pharmacological* **Rotigotine**	Increased astrocyte proliferation through the overexpression of the Nrf2 signaling pathway.	Reduction in dopaminergic neurodegeneration.	α-syn transgenic mutant mice (A53T).	[108,109,110]
**NLY01**	Regulation of inflammatory signaling mediated by NF-ΚB.	Modulation of mitochondrial biogenesis and microglial inflammation, as well as enhanced autophagy.	Parkinsonian mice.	[103]
**Conversion of astrocytes into DA neurons**	Reprogramming of striatal and VM astrocytes into DA neurons.	Increase in neuronal maturity, increase in levels of specific VM markers, greater resistance to cytokines, improvement of motor deficits, and restoration of DA levels in VM.	Culture of human astrocytes, PD mice 6-OHDA.	[122,123,124]
**Transplant of VM astrocytes**	Grafting VM cultured astrocytes into the SNpc.	Nigrostriatal neuronal rescue, greater DA capture, improvement of motor deficits, elimination of toxic α-syn aggregates, and decrease in DA neurodegeneration.	Aged Parkinsonian mice and mice with hemi-Parkinson’s.	[123,125,126]
**Therapies Targeting α-Syn**				
**Therapies**	**Effects in α-syn**	**Findings in the PD**	**Experimental Approach**	**References**
**Natural small-molecule compounds**	Suppression of neurotoxicity of α-syn oligomers, blocking of α-syn aggregation processes, and prevention of the formation of α-syn oligomers.	Reduction in motor deficits and neuroprotective effects, and a significant reduction in toxic α-syn fibrils.	Parkinsonian mice *in vitro*.	[128,129,130]
**α** **-syn-specific antibodies**	Neutralization of toxic α-syn aggregates, reduced intracellular aggregation, and decreased accumulation of toxic α-syn oligomers.	Deceleration of neurodegeneration and reduction in motor deficits.	PD transgenic mice, PD mice.	[133,134,135]

α-syn, alpha-synuclein; 6-OHDA, 6-hydroxydopamine; DA, dopamine; PD, Parkinson’s disease; SNpc, substantia nigra pars compacta; VM, ventral midbrain.

## Data Availability

Not applicable.
